# An Association Study of Germline Variants in Bladder Cancer-Related Genes with the Prognosis of Non-Muscle Invasive Bladder Cancer

**DOI:** 10.3233/BLC-220076

**Published:** 2023-03-31

**Authors:** Jasper P. Hof, Sita H. Vermeulen, Antoine G. van der Heijden, Gerald W. Verhaegh, Lars Dyrskjøt, James W.F. Catto, Lourdes Mengual, Richard T. Bryan, Neil E. Fleshner, Lambertus A.L.M. Kiemeney, Tessel E. Galesloot

**Affiliations:** a Department for Health Evidence, Radboud University Medical Center, Nijmegen, The Netherlands; b Department of Urology, Radboud University Medical Center, Nijmegen, The Netherlands; c Department of Molecular Medicine, Aarhus University Hospital, Aarhus, Denmark; d Department of Clinical Medicine, Aarhus University, Aarhus, Denmark; e Academic Urology Unit, University of Sheffield, Sheffield, UK; f Department and Laboratory of Urology, Universitat de Barcelona, Barcelona, Spain; g Institute of Cancer & Genomic Sciences, Bladder Cancer Research Centre, University of Birmingham, Birmingham, UK; h Department of Urology, Princess Margaret Cancer Centre, Toronto, ON, Canada

**Keywords:** Bladder cancer, recurrence, progression, candidate gene analysis, genetic association study

## Abstract

**BACKGROUND:**

Various germline genetic variants are associated with the prognosis of non-muscle invasive bladder cancer (NMIBC). Germline variants in genes frequently somatically mutated in bladder cancer have not been studied thoroughly in relation to risk of recurrence or progression in NMIBC.

**OBJECTIVE:**

To identify germline DNA variants in bladder carcinogenesis-related genes associated with recurrence or progression in NMIBC.

**METHODS:**

We analysed associations between single-nucleotide polymorphisms (SNPs) and NMIBC recurrence and progression using data from the Nijmegen Bladder Cancer Study (NBCS, 1,443 patients). We included 5,053 SNPs within 46 genes known to have mutation, overexpression or amplification in bladder cancer. We included all recurrences in the statistical analysis and performed both single variant analysis and gene-based analysis. SNPs and genes that showed significant or suggestive association (false discovery rate P value < 20%) were followed-up in independent cohorts for replication analysis, through eQTL analysis and tests for association of tumour expression levels with NMIBC recurrence and progression.

**RESULTS:**

Single variant analysis showed no statistically significant associations with recurrence or progression. In gene-based analysis, the aggregate effect of the 25 SNPs in the Cyclin D1 gene (*CCND1*) was statistically significantly associated with NMIBC recurrence (P_unadj_ = 0.001, P_FDR_ = 0.046), but not with progression (P_unadj_ = 0.17, P_FDR_ = 0.54). Validation analysis in independent cohorts did not confirm the association of *CCND1* with NMIBC recurrence.

**CONCLUSIONS:**

We could not identify reproducible associations between common germline variants in bladder carcinogenesis-related genes and NMIBC recurrence or progression.

## INTRODUCTION

Approximately 75% of bladder cancer patients are diagnosed with non-muscle invasive bladder cancer (NMIBC) [1], which has a favourable 5-year disease-specific survival of 85–90%, but is characterized by a high risk of multiple tumour recurrences and risk of progression to muscle-invasive bladder cancer (MIBC) [2]. As a consequence, patients need regular follow-up cystoscopies for surveillance and treatment of recurrences (transurethral resection of bladder tumour (TURBT)). The rate of recurrence amongst NMIBC patients varies widely, with some patients experiencing many and frequent recurrences, whilst others remain recurrence-free for the rest of their lives [2, 3].

Tumour multiplicity, size and prior recurrence have been reported to be the most important predictors for NMIBC recurrence [3], whereas stage, associated carcinoma *in situ* (CIS) and grade are the most important predictors for progression [4]. There are also suggestions that NMIBC prognosis can be affected by lifestyle choices [5].

The role of germline genetic variants in NMIBC prognosis has gained attention over the last years. Genetic variation in the sonic hedgehog pathway has been reproducibly linked to NMIBC recurrence risk [6]. A study by Grotenhuis et al. tested associations between germline genetic variants previously reported to be associated with NMIBC prognosis, however, none of the 114 evaluated variants could be replicated after adjustment for multiple testing [7]. More recently, we conducted a meta-analysis of genome-wide association studies (meta-GWAS) to detect germline DNA variants associated with risk of recurrence or progression in NMIBC [8]. We found variants in *G2E3* and *SCFD1* that were genome-wide significantly associated with recurrence-free survival (RFS) and twelve other SNPs showed suggestive association with RFS. We subsequently confirmed that expression of *SCFD1* was associated with RFS in data from the UROMOL study [9].

In recent years, several studies have described the somatic mutation profile of NMIBC [9, 10]. *FGFR3* and *TERT* are the most frequently mutated oncogenes for bladder cancer [10, 11]. Other genes that are frequently mutated in NMIBC are RAS-genes and *PIK3CA* [12, 13]. Note that germline SNPs in both *FGFR3* and *TERT* have been associated with bladder cancer risk [14, 15]. With regard to NMIBC outcome, a common germline genetic variant in the *TERT* promotor, rs2853669, may modify the effects of somatic mutations in the *TERT* promotor region on RFS [16]. Somatic mutations in *PIK3CA* have been associated with reduced risk of recurrence and improved disease-specific survival [17, 18]. Ward et al. reported associations between mutations in *RXRA*, *RHOB* and *TERT* with recurrence-free survival [13]. Having a mutation in at least one of the genes *FGFR3*, *TP53*, *PIK3CA*, *CKN2A*, *HRAS*, *KRAS*, *ERBB2*, *VHL*, *MLL* or *MET* was associated with increased risk of progression [19], and gene expression levels of *RXRA* and *FGFR3* were associated with recurrence-free survival [20]. Nevertheless, in our meta-GWAS for NMIBC prognosis, common germline variants in genes that often show somatic mutations in bladder cancer were not among the top signals. Also, these genes have not been thoroughly studied in germline candidate gene studies.

Here, we investigated the association of NMIBC recurrence and progression with common germline DNA variants in 46 genes that exhibit somatic mutation, amplification or overexpression in bladder cancer. We included all potential recurrences that a patient might experience in statistical analysis of recurrence risk, to increase power and avoid the bias of only including the initial recurrence (usually reported as recurrence-free survival (RFS)) [21].

## MATERIALS AND METHODS

### Study population

Patient data were retrieved from the Nijmegen Bladder Cancer Study (NBCS). In the NBCS, patients diagnosed with bladder cancer in seven hospitals in the mid-east of the Netherlands were identified through the National Cancer Registry held by the Netherlands Comprehensive Cancer Organisation (IKNL). In 2007, the NBCS started with the identification of urothelial bladder carcinoma (UBC) patients aged under 75 years and diagnosed between 1995–2006 and invited them to participate. Three additional cohorts of patients diagnosed later (2006–2008, 2008-2009 and 2009-2010) were invited in January 2009, November 2010 and February 2012 respectively. In total, 66% of the invitees participated. The date of diagnosis, stage, grade and focality of the primary tumour and all recurrent tumours were recorded. The study was approved by the research ethics committee (CMO Arnhem-Nijmegen, approval number 2005/315). All participants provided informed consent.

### Genotyping and quality control

All patients were genotyped using Illumina OmniExpress-12 and -24 chips and imputed to higher SNP density using 1000 Genomes and Genome of the Netherlands [22] as reference panels. After imputation, we excluded SNPs with 1) a minor allele frequency (MAF) < 0.05, 2) Hardy-Weinberg Equilibrium *P*-value < 10^-5^, or 3) an IMPUTE2 imputation info score < 0.8. More details about the imputation and quality control pipeline are provided in supplement 1.

### Candidate genes

We used three gene panels to select candidate bladder cancer genes: 1) the UROseek panel, which comprises 11 genes that include the most common genetic alterations in bladder cancer [19]; 2) a 29-gene panel with genes involved in bladder carcinogenesis because of activating mutations or overexpression, which is derived from the analysis of The Cancer Genome Atlas and recent literature [20]; and 3) a 23-gene panel to detect somatic mutations that are involved in UBC pathogenesis [13]. The three gene panels comprise 46 unique autosomal genes, four of which were included in all three gene panels (*HRAS*, *ERBB2*, *FGFR3*, *PIK3CA*) (see supplemental information for all genes). All SNPs located in the genes and its 10 kb surrounding region that met the inclusion criteria were extracted from the genetic data (gene locations based on NCBI build 37.p13), resulting in 5,053 SNPs (647 directly genotyped, 4,406 imputed). The median number of SNPs in the genes was 76 (range: 2–412).

### Outcome definitions

The start of the follow-up is marked by the date of the primary TURBT. Recurrences are defined as a new, histologically confirmed bladder or prostatic urethra tumour following at least one tumour-negative urethrocystoscopy (UCS) or following two surgical resection attempts for the previous bladder tumour (usually a TURBT and radical re-TURBT). The date of progression was defined as the first date at which there was a transition from low-grade to high-grade disease, or an increase in T stage, N stage or M stage. Cystectomy for therapy-resistant or “uncontrollable” disease was also coded as progression. More details about definitions are included in the supplemental information.

### Statistical analysis

#### Choice of statistical model

Progression-free survival was analysed using a Cox proportional hazards (CoxPH) model. We selected the Gap Time - Unrestricted (GT-UR) model for analysis of the associations of the SNPs with all NMIBC recurrences. The GT-UR model is an extension of the CoxPH model that is commonly used to study survival or prognostic outcomes. The difference between both models is that the GT-UR model can model all recurrences that a patient might experience, whereas the CoxPH model can only model the time to first recurrence and ignores all subsequent recurrences. A lognormal frailty term was included in the GT-UR model to account for the fact that the recurrences that occur within a patient are correlated.

The GT-UR model tests for associations between SNPs and recurrences using gap time as a time scale. This means that the time between the removal of the previous tumour and the subsequent recurrence is used as outcome, essentially ‘resetting’ the time to zero after every recurrence that a patient experiences.

The GT-UR model is based on the same assumptions as the CoxPH model. In addition, the GT-UR model assumes a constant effect of genetic variants on recurrence rate for all recurrences.

In the analysis of recurrences, the hazard ratio (HR) derived from a GT-UR model has a different interpretation compared to the HR from a CoxPH model. The HR from the GT-UR model denotes the modified recurrence risk for any recurrence from the previous recurrence/primary tumour onwards, whereas the HR from the CoxPH model denotes the modified recurrence risk for the first recurrence, from the diagnosis of the primary tumour onwards. The latter interpretation also holds for hazard ratios obtained from the CoxPH model in analyses of progression. Note that risk of progression can not be analyzed using the GT-UR model, since there can only be one event of progression.

The coxph function of the R package ‘survival’ v3.2-13 was used for analysis of progression, the coxme function of the R package ‘coxme’ v2.2-16 was used for analysis of recurrences. More information on model options in recurrent event analysis and our model selection procedure can be found in the supplemental information.

#### Single SNP analysis

We performed SNP analysis (i.e. test each SNP individually for association) based on the additive genotype model where the presence of an alternative allele is counted as 1, i.e. patients homozygous for the reference allele are classified as 0, heterozygous patients as 1, and patients homozygous for the alternative allele as 2. We also investigated the potential effect of clinical variables, namely age, sex, stage and grade of recurrences. The adjustment for these variables did not change the effect estimates of SNPs on NMIBC recurrence or progression, so we did not include these covariates in the final analysis. To adjust for multiple testing, a false discovery rate threshold (FDR) of 5% was used.

#### Gene-based analysis

We also performed gene-based analysis to test the aggregated association between all SNPs that are present within a single gene with recurrence and progression. First, we constructed - for every gene separately - the principal components based on all SNPs that are present in that gene and the 10kb surrounding region. Next, we selected the top principal components that explained > 99.9% of the genetic variation in the gene. These principal components summarize the information that is present in all the SNPs within a gene. These were then modelled together in the CoxPH model or GT-UR model for their effect on NMIBC progression or recurrences, respectively. For statistical significance testing, we used a likelihood ratio test (LRT) to assess the effect of genetic variation within the gene on tumour recurrence or progression. To adjust for multiple testing, again an FDR of 5% was used.

### Validation analysis

SNPs and genes that were statistically significantly or suggestively associated with NMIBC recurrence or progression (FDR < 20%) were validated in independent cohorts. Only one gene, *CCND1*, fulfilled this condition in the analysis of recurrences. For this gene, we tested the association of gene expression in tumour tissue with recurrence-free survival in data from the UROMOL study [9], consisting of 535 NMIBC patients.

In addition, we investigated the associations of SNPs in *CCND1* with tumour gene expression (eQTL analysis). The SNP data of *CCND1* in the UROMOL cohort comprised 29 SNPs within the 10kb window of *CCND1* in 320 NMIBC patients. In total, 19 SNPs were included in analysis after filtering out SNPs at a MAF less than 0.05, and 318 NMIBC patients remained eligible for analysis after removing patients with a missing genotype rate greater than 5%. The 19 SNPs in *CCND1* were tested for association with *CCND1* expression using a linear regression, and their statistical significance was assessed at an FDR adjusted P value smaller than 0.05. The aggregate effect of germline genetic variation in *CCND1* on *CCND1* expression was also tested using a linear principal component regression, which includes the top principal components that explain > 99.9 percent of the genetic variation in *CCND1*.

Finally, we validated the association of *CCND1* with recurrence-free survival using summary statistics from our recently published meta-GWAS on NMIBC prognosis, which included data from the following cohorts: the Bladder Cancer Prognosis Programme (BCPP, Birmingham; N = 684), two cohorts from the Genito-Urinary BioBank (GUB-1, GUB-2, Toronto, Canada; N = 353 and 432, respectively), and biobanked case series from the University of Sheffield (Sheffield, UK; N = 244) and the Hospital Clínic of Barcelona (Barcelona, Spain; N = 238) [8]. We excluded results of the NBCS from the meta-GWAS results to achieve independent validation. The association between *CCND1* and RFS was assessed using gene-based analysis performed in MAGMA software, as available in the web-based platform FUMA [23, 24]. The analysis in MAGMA included 9 SNPs in *CCND1* in 1,271 individuals in total.

## RESULTS

### Patient characteristics

**Fig. 1:**
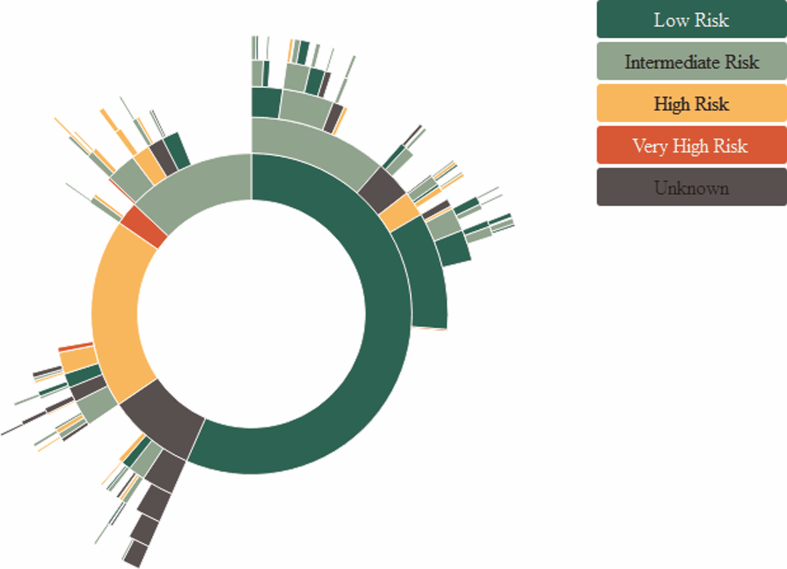
Recurrence patterns in individuals per prognostic group in the Nijmegen Bladder Cancer Study. Every layer away from the centre represents a new recurrence, the circle in the middle represents the characteristics of the primary tumour. Prognostic risk groups were assessed using a modified version of EAU prognostic risk categories, as not all clinical data were available [2]. Risk groups are defined in supplemental document 1.

In total, 1,443 patients who experienced 1,864 recurrences and 167 progression events were included in the analysis. Patient and tumour characteristics at primary diagnosis, for progression and for the first to fourth recurrence are displayed in Table 1. Median follow-up time (*i.e.* time between TURBT of the primary tumour and end of follow-up) was 4.1 years (interquartile range: 2.6–6.7 years). In the NBCS, the 1-year Kaplan Meier (KM) risk of progression was 3%; the 5-year KM risk of progression was 14%. In total, 709 patients reported at least one recurrence before they reached the end of follow-up. Among them, 392 patients reported a total of 1,155 recurrences after the first recurrence, which are included in our analyses but would have been ignored in a traditional CoxPH model. The 1-year Kaplan Meier (KM) risk of first recurrence after primary TURBT was 24%; the 5-year Kaplan Meier risk of first recurrence was 53%. The second recurrence had a 1-year KM risk of 33% and a 5-year KM risk of 65%, the third recurrence had a 1-year KM risk of 39% and a 5-year KM risk of 78%. These recurrence risks are based on time from previous recurrence onwards and are based on the study population that had one resp. two recurrences. These populations are frailer to tumour recurrences, which leads to higher recurrence risks. An overview of recurrence patterns stratified into prognostic risk groups is displayed in Fig. 1. Note that patients in high-risk prognostic groups underwent more radical therapies (*e.g.* cystectomy) compared to the low-risk groups, which lead to shorter follow-up time and less recurrences.

**Table 1:** Patient and tumour characteristics of NMIBC patients in the Nijmegen Bladder Cancer Study for the total study population and for the subgroups of patients that experienced at least 1, 2, 3 or 4 recurrences

Nijmegen Bladder Cancer Study		Primary	Progression	Rec 1	Rec 2	Rec 3	Rec 4
		(N = 1,443)	(N = 167)	(N = 709)	(N = 392)	(N = 242)	(N = 160)
Male Gender		1191 (83)	138 (83)	589 (83)	321 (82)	197 (81)	127 (80)
Median Age^1^ (range in years)		64 (25–91)	64 (31–88)	64 (29–85)	62 (29–84)	62 (29 –82)	60 (29 –82)
Smoking status^1^	Never smoker	245 (17)	25 (15)	103 (15)	57 (15)	38 (16)	26 (16)
	Ever smoker	1091 (76)	127 (76)	518 (73)	275 (70)	166 (69)	108 (68)
	Unknown	107 (7)	15 (9)	88 (12)	60 (15)	38 (16)	26 (16)
Stage tumour^2^	Ta	1018 (71)	81 (49)	395 (56)	229 (58)	138 (57)	86 (54)
	T1	339 (23)	66 (40)	50 (7)	22 (6)	14 (6)	12 (8)
	CIS	54 (4)	12 (7)	71 (10)	24 (6)	11 (5)	8 (5)
	Unknown	32 (2)	8 (5)	193 (27)	117 (30)	79 (33)	54 (34)
Concomitant CIS	No	1318 (91)	132 (79)	494 (70)	264 (67)	153 (63)	102 (64)
	Yes	93 (6)	27 (16)	22 (3)	11 (3)	10 (4)	4 (3)
	Unknown	32 (2)	8 (5)	193 (27)	117 (30)	79 (33)	54 (34)
Grade tumour^3^	Low grade	940 (65)	84 (50)	337 (48)	196 (50)	113 (47)	73 (46)
	High grade	465 (32)	77 (46)	172 (24)	79 (20)	49 (20)	33 (21)
	Unknown	38 (3)	6 (4)	200 (28)	117 (30)	80 (33)	54 (34)

^1^Age and smoking status are based on age and smoking status at primary diagnosis. ^2^In case of Ta/T1 with concomitant CIS, only Ta/T1 is included in stage. ^3^Low grade denotes WHO 1973 differentiation grade 1 or 2, WHO/ISUP 2004 PUNLMP/low grade or Bergkvist grade 1/2a, high grade denotes WHO 1973 differentiation grade 3, WHO/ISUP 2004 high grade, or Bergkvist grade 2b/3.

### Single SNP analysis

Both in recurrence and progression analyses, none of the SNPs reached the multiple testing adjusted threshold for statistical significance. The ten most strongly associated loci based on statistical significance, summarized by the strongest associated SNP in that region, are displayed in Tables 2 and 3. SNP rs114873844 in *ELF3* showed the strongest association with NMIBC recurrences (HR = 0.68 (95% confidence interval [CI] 0.54,0.86), P_FDR_ = 1.00, P_unadj_ = 0.0013); SNP rs7586307 in *NFE2L2* showed the strongest association with progression (HR = 1.72 (95% CI 1.25, 2.37), P_FDR_ = 1.00, P_unadj_ = 0.0007).

**Table 2:** Results of the ten SNPs with strongest association in single SNP analysis with NMIBC recurrences in the Nijmegen Bladder Cancer Study

Chr	SNP ID	Position	A1	A2	MAF	Gene	Beta	HR	95% CI	SE	P_FDR_	P_unadj_
1	rs114873844	201979370	G	A	0.06	*ELF3*	–0.39	0.68	(0.54, 0.86)	0.12	1.00	0.0013
2	rs11889962	20645915	A	G	0.06	*RHOB*	0.35	1.41	(1.14, 1.75)	0.11	1.00	0.0014
10	rs41282876	129899482	T	A	0.10	*MKi67*	0.25	1.28	(1.09, 1.50)	0.08	1.00	0.0029
10	rs77393382	129928636	T	C	0.11	*MKi67*	0.22	1.25	(1.07, 1.47)	0.08	1.00	0.0062
19	rs3916898	45854330	A	C	0.07	*ERCC2*	0.26	1.30	(1.07, 1.58)	0.10	1.00	0.0071
12	rs10876869	56467865	C	G	0.40	*ERBB3*	0.14	1.15	(1.04, 1.28)	0.05	1.00	0.0084
3	rs4135294	12466715	G	A	0.15	*PPARG*	0.18	1.20	(1.05, 1.38)	0.07	1.00	0.0092
5	rs2736109	1296759	C	T	0.43	*TERT*	0.15	1.16	(1.04, 1.29)	0.06	1.00	0.0100
7	rs6970262	55259763	A	G	0.64	*EGFR*	0.14	1.15	(1.03, 1.29)	0.06	1.00	0.0110
3	rs9833097	12478817	G	A	0.10	*PPARG*	0.21	1.24	(1.05, 1.46)	0.08	1.00	0.0110

A1: Reference allele, A2: Alternative allele, MAF: Minor Allele Frequency, HR: Hazard ratio, CI: Confidence Interval, SE: Standard Error, P_FDR_: False Discovery Rate-corrected P value, P_unadj_: Unadjusted P value.

**Table 3:** Results of the ten SNPs with strongest association in single SNP analysis with NMIBC progression in the Nijmegen Bladder Cancer Study

Chr	SNP ID	Position	A1	A2	MAF	Gene	Beta	HR	95% CI	SE	P_FDR_	P_unadj_
2	rs7586307	178138926	C	T	0,10	*NFE2L2*	0,54	1,72	(1.25, 2.37)	0,16	1.00	0.00079
10	rs77393382	129928636	T	C	0,11	*MKi67*	0,48	1,62	(1.19, 2.21)	0,16	1.00	0.002
3	rs1642743	10190467	T	C	0,38	*VHL*	0,36	1,43	(1.14, 1.80)	0,12	1.00	0.002
6	rs2376620	36649593	A	G	0,17	*CDKN1A*	0,41	1,51	(1.16, 1.97)	0,14	1.00	0.0024
12	rs11171744	56503127	G	C	0,12	*ERBB3*	0,51	1,67	(1.20, 2.33)	0,17	1.00	0.0024
11	rs655089	69448575	T	G	0,46	*CCND1*	0,33	1,38	(1.11, 1.72)	0,11	1.00	0.0035
17	rs12951053	7577407	A	C	0,09	*TP53*	0,46	1,59	(1.16, 2.17)	0,16	1.00	0.0042
17	rs17883048	7570956	G	A	0,05	*TP53*	0,58	1,79	(1.18, 2.72)	0,21	1.00	0.0062
10	rs117040846	5827619	G	A	0,05	*GDI2*	0,59	1,81	(1.17, 2.78)	0,22	1.00	0.0072
11	rs11603541	69472373	C	G	0,11	*CCND1*	–0,60	0,55	(0.35, 0.85)	0,23	1.00	0.0073

A1: Reference allele, A2: Alternative allele, MAF: Minor Allele Frequency, HR: Hazard ratio, CI: Confidence Interval, SE: Standard Error, P_FDR_: False Discovery Rate-corrected P value, P_unadj_: Unadjusted P value.

### Gene-based analysis

**Fig. 2:**
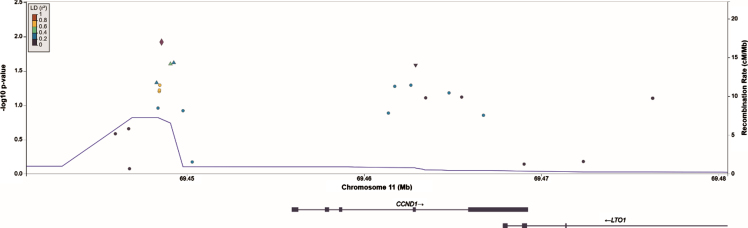
Regional association plot of SNPs in and nearby *CCND1* constructed in LocusZoom. The SNP in *CCND1* that shows the strongest association with NMIBC recurrences, rs655089, is marked by the purple diamond.

The ten genes with the lowest P values in recurrence and progression analysis are displayed in Tables 4 and 5, respectively. SNPs in the *CCND1* locus collectively showed the strongest evidence for association and reached statistical significance for recurrence (LRT = 43.8, P_FDR_ = 0.046), but not for progression (LRT = 24.7, P_FDR_ = 0.54). *ERBB3*, *FGFR3*, *CDKN2A*, *ERCC2* and *KRAS* had unadjusted P values < 0.05 in recurrence analysis, but when corrected for false discovery rate had P values > 0.2 and were thus not carried forward for validation. Similarly, *PPARG* and *KRAS* were no longer significant after correction for false discovery rate in progression analyses. Note that many genes have a similar false-discovery corrected P value as a result of the Benjamini-Hochberg procedure. A regional association plot of the *CCND1* gene region is shown in Fig. 2.

**Table 4:** Results of the gene-based analysis in which germline genetic variation within a gene was tested for association with NMIBC recurrence in the Nijmegen Bladder Cancer Study

Gene	Number of SNPs	DF	LRT	P_FDR_	P_unadj_
*CCND1*	25	19	43.82	0.046^*^	0.001^*^
*ERBB3*	35	18	32.51	0.245	0.019
*FGFR3*	20	17	30.25	0.245	0.025
*CDKN2A*	54	31	47.38	0.245	0.030
*ERCC2*	73	40	58.32	0.245	0.031
*KRAS*	177	45	64.13	0.245	0.032
*MET*	126	49	64.16	0.459	0.072
*RHOB*	43	19	27.76	0.459	0.088
*RXRA*	237	79	95.57	0.459	0.099
*PRKCI*	106	34	44.47	0.459	0.108

DF: Degrees of Freedom (DF) in the likelihood ratio test, LRT: Likelihood Ratio Test (LRT) statistic. P_FDR_: False Discovery Rate-corrected P value, P_unadj_: Unadjusted P value. ^*^P values in bold exceeded the threshold for statistical significance.

**Table 5:** Results of the gene-based analysis in which germline genetic variation within a gene was tested for association with NMIBC progression in the Nijmegen Bladder Cancer Study

Gene	Number of SNPs	DF	LRT	P_FDR_	P_unadj_
*PPARG*	290	73	98,15	0,539	0,027
*ERBB3*	35	18	30,87	0,539	0,030
*KRAS*	177	45	63,29	0,539	0,037
*ZNF703*	2	2	5,16	0,539	0,076
*MDM2*	77	29	38,55	0,539	0,111
*FBXW7*	82	33	42,46	0,539	0,125
*MKi67*	179	64	76,76	0,539	0,132
*MDM4*	217	52	62,80	0,539	0,145
*CDKN2A*	54	31	39,03	0,539	0,153
*PABPC1*	65	20	26,09	0,539	0,163

DF: Degrees of Freedom (DF) in the likelihood ratio test, LRT: Likelihood Ratio Test (LRT) statistic. P_FDR_: False Discovery Rate-corrected P value, P_unadj_: Unadjusted P value.

The SNP with the strongest association in *CCND1* in single SNP analysis was rs655089 (HR = 1.14 (95% CI 1.03, 1.26), P_FDR_ = 1.00, P_unadj_ = 0.012), which is located upstream of *CCND1.* SNP rs655089 was the main driver of the gene-based association for *CCND1* with recurrence: no other SNP in *CCND1* exceeded the nominal significance threshold (P_unadj_  < 0.05) in single SNP analysis when rs655089 was included as a covariate.

### Validation analysis

*CCND1* tumour expression was not statistically significantly associated with risk of recurrence in NMIBC patients from the UROMOL cohort (HR = 0.96 (95% CI 0.89,1.04), *P* = 0.35). The SNP with the strongest association in *CCND1*, rs655089, is located upstream of *CCND1* and could act on *CCND1* expression in tumour tissue through transcription factor binding. However, we did not reveal statistically significant associations between SNPs in *CCND1* and tumour expression of *CCND1* in eQTL analysis (Table 6). Also, the aggregate effect of all SNPs in *CCND1* was not associated with *CCND1* tumour expression in a likelihood-ratio test (*P* = 0.33). No statistically significant association was observed for the aggregated effect of germline genetic variants in *CCND1* and recurrence-free survival in the meta-GWAS for NMIBC recurrence (*P* = 0.65).

**Table 6:** Result of eQTL analysis of 19 SNPs in *CCND1* in the UROMOL study

SNP ID	Position	A1	A2	MAF	Beta	SE	*P* value
rs11824610	69446331	G	A	0.06	0.43	0.22	0.05
rs11826558	69446766	C	T	0.07	0.41	0.20	0.04
rs653810	69448294	G	A	0.38	0.02	0.11	0.87
rs654240	69448373	C	T	0.41	–0.02	0.10	0.88
rs654648	69448445	G	A	0.41	0.04	0.11	0.68
rs655089	69448575	T	G	0.42	0.07	0.11	0.52
rs2450254	69449784	A	T	0.38	–0.03	0.11	0.81
rs35654475	69452339	–	CCAG	0.50	0.06	0.10	0.58
rs117459970	69452710	C	G	0.50	0.06	0.10	0.58
rs77290390	69453506	G	A	0.11	0.01	0.15	0.94
rs187210029	69457293	C	T	0.49	0.09	0.10	0.38
rs1352075	69461182	C	T	0.13	–0.08	0.16	0.62
rs55816909	69463479	G	C	0.49	–0.04	0.10	0.67
rs3918297	69464793	A	G	0.46	–0.09	0.10	0.35
rs3212870	69465507	A	C	0.46	–0.10	0.10	0.33
rs3212877	69465681	G	A	0.43	–0.10	0.10	0.33
rs183501442	69466115	A	C	0.49	–0.13	0.10	0.20
rs34193475	69466737	G	C	0.35	–0.10	0.10	0.33

A1: Reference allele, A2: Alternative allele, MAF: Minor Allele Frequency, Beta: Linear regression coefficient, SE: Standard Error.

## DISCUSSION

Our study investigated the relationship between germline genetic variants in known bladder cancer predisposition genes with bladder cancer prognosis. While somatic alterations in these genes are known to contribute to the development of bladder cancer and some of them were found to play a role in bladder cancer prognosis, the effect of germline variation in these genes on recurrence or progression has not been investigated before in depth. We identified a statistically significant association between germline genetic variation in gene *CCND1* and NMIBC recurrence in a recurrent event analysis, which includes all recurrences of NMIBC patients in statistical analysis. However, this association could not be confirmed using association analyses of germline *CCND1* variants, *CCND1* tumour gene expression, and recurrence-free survival in additional independent cohorts. We did not find statistically significant associations for germline variation in any of the other candidate genes with recurrence or progression.

It is possible that our validation analysis has resulted in a false-negative finding. First of all, it could be that our validation analyses were underpowered compared to our discovery analyses. The power of our discovery analyses was optimized by: 1) performing analyses in the NBCS cohort, the currently largest prognostic cohort on NMIBC outcome; 2) performing a recurrent event analysis instead of a time-to-first recurrence analysis, thereby including all potential recurrence a patient might experience [21]; and 3) including a gene-based analyses based on individual-level data [23]. The recurrent event analysis resulted in 80% power to identify SNPs with minor allele frequency (MAF) 0.3 and HR 1.37 using a Bonferroni corrected P value significance threshold of 0.05/5,053 = 9.9×10^-6^, whereas a time-to-first event analysis would have 80% power to identify SNPs with MAF 0.3 and HR 1.46. In addition, the validation cohorts that we used were of individually smaller sample size and did not register all recurrences a patient might experience, thus only enabling a time-to-first recurrence analysis. This caused a reduced power in our validation analyses and potentially false negative results. On the other hand, the association between *CCND1* and total NMIBC recurrence risk was mainly driven by the effect on first recurrence: when we restricted our gene-based analysis to time until first recurrence only in a CoxPH model, SNP variation in *CCND1* was associated with RFS at a P_unadj_ = 0.0087 (P_FDR_ = 0.15) using a likelihood-ratio test.

**Fig. 3:**
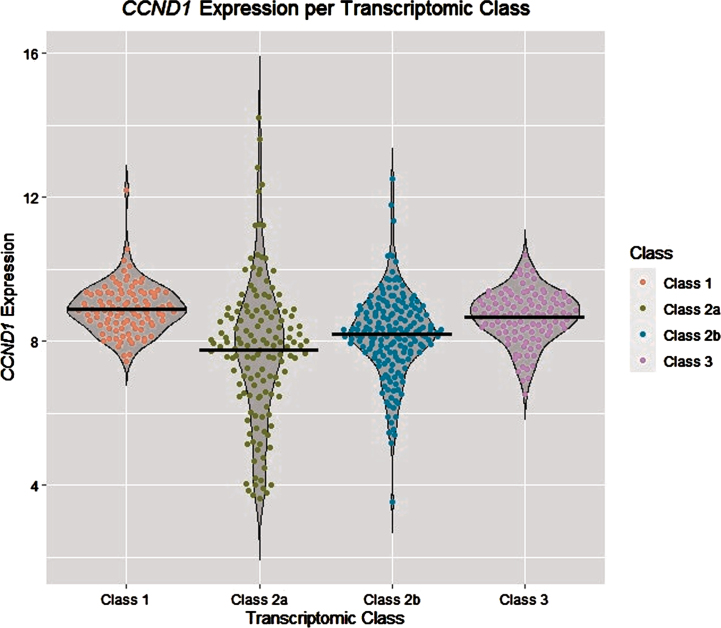
Expression levels of *CCND1* in tumours from different transcriptomic classes in publicly available data from UROMOL [9]. The black horizontal lines mark the mean expression levels per class.

Secondly, a true association between tumour expression of *CCND1* and RFS could have been masked by the presence of interaction effects between *CCND1* and other genes. In data from UROMOL, we observed that *CCND1* tumour expression differed in the four transcriptomic classes described in the UROMOL study (Fig. 3) [9], which were prognostic for RFS and progression-free survival in NMIBC: patients with primary tumours in transcriptomic classes 1 and 3 had low recurrence- and progression rates compared to patients in classes 2a and 2b. Thus, there might be epistasis between *CCND1* and other genes included in the transcriptomic classes, which might have masked the association between *CCND1* and RFS. In addition, we note that *CCND1* was included in the gene panel by Le Goux et al. because of recurrent amplifications in bladder cancer. This amplification could affect *CCND1* tumour expression, which makes it more difficult to compute direct associations between SNP variation and *CCND1* expression in eQTL analysis.

Finally, the coverage of common genetic variation was not optimal for all candidate genes. The median number of SNPs per gene was 76, but for some genes our data contained few SNPs, e.g. ZNF703 and SOX4 contained < 10 SNPs. This might have led to lack of coverage of the genetic variation in these genes and potentially false-negative results.

We did not observe any statistically significant association for progression. Compared to recurrence, the gene panels that we used to select candidate genes also reported relatively few associations with PFS: only *RXRA* overexpression, and having a mutation in any UROseek gene were associated with PFS [13, 19, 20]. This could be due to the small number of progression cases in these panels: the UROseek panel only reported 21 cases of progression, whereas the studies by Le Goux and Ward only reported 10 and 25 cases of progression, respectively [13, 19, 20]. The candidate genes were also not amongst the top signals of our recent genome-wide association study on NMIBC prognosis [8]. However, our analyses may have missed associations for progression due to limited power: for MAF 0.3 and a multiple testing corrected significance threshold of 9.9×10^-6^, our progression analysis had 80% power to detect SNPs with HR 1.82, whereas we had a 80% power to detect SNPs with HR of 1.37 in our recurrence analysis. Finally, low coverage of common genetic variation could also have led to false-negative findings, like we described for recurrence.

Notably, recurrent event analysis gives more weight to patients who experienced more recurrences, because the analyses are performed at the level of the recurrence. It could be that this approach prioritizes effects in patients with frequently recurring low-risk tumours, which could diminish the generalizability of the results to the total NMIBC population. This is not the case in our study, as we observed that patients with frequently recurring tumours were not at lower risk of progression. First of all, the patient characteristics in Table 1 show a similar distribution of stage and grade for the first to fourth recurrence, which suggests a similar risk profile for patients who experienced multiple recurrences vs. patients with no recurrence. In addition to this, we tested the correlation between individual risk of recurrences and progression following the methodology by Balan et al. [25]. Patients who experienced more recurrences had a slightly increased risk of progression to MIBC (*p* = 0.03), which suggests that the analysis of all recurrences does not prioritize low-risk disease.

Our study has some strengths and limitations. A main strength of our work is the analysis of all NMIBC recurrences, instead of analysing only recurrence-free survival. Another strength is our study population: the NBCS is a population-based cohort with a large sample size, clinical data were carefully cleaned in consultation with urologists and experts in bladder cancer, and our genotype data had a high SNP density due to imputation. We acknowledge some limitations: our study did not cover low-frequency or rare genetic variation and might also have missed some common SNPs that were not measured and/or imputed with low precision; and our study population is at risk of prevalent case bias due to the delay between NMIBC diagnosis and invitation to the NBCS.

In conclusion, we identified a statistically significant association between germline DNA variation in *CCND1* and NMIBC recurrences, however, this association was not validated in additional independent cohorts. None of the other genes related to bladder-carcinogenesis were statistically significantly associated with NMIBC recurrence or progression. We recommend to repeat this work once larger sample sizes are available.

## Supplementary Material

Supplementary Material
