# Supplementary material for: An Association Study of Germline Variants in Bladder Cancer-Related Genes with the Prognosis of Non-Muscle Invasive Bladder Cancer
Source: Bladder Cancer. 2023 Mar 31;9(1):59–71. doi: 10.3233/BLC-220076 (PMC11181778; doi:10.3233/BLC-220076)
Supplement: Supplementary Material [file blc-9-blc220076-s001.docx]

**Supplemental information**

Genotyping and imputation

Genotyping, imputation and quality control of the NBCS genotype data were described before [1]. In summary, DNA samples were genotyped with Illumina OmniExpress-12 or -24 chips and subsequently imputed to a higher SNP density using combined 1000 Genome Project phase 1 integrated version 3 and Genome of the Netherlands 4 (GoNL4) panel as a reference (hg19) in SHAPEIT and IMPUTE2 software. SNPs were excluded prior to imputation if (a) minor allele frequency (MAF) < 1%; (b) deviation from Hardy-Weinberg equilibrium (p-value < 1.0 x 10^-4^); SNP yield < 95%. Furthermore, individuals with a call rate <95% were removed from the data. This resulted in 577,389 SNPs for imputation.

Imputation resulted in 20,011,335 single nucleotide variants (SNVs). Post-imputation quality control consisted of: (a) check for gender mismatch: individuals with different sex in genotype data as compared to clinical data were removed; (b) ethnicity check: samples were projected on HapMap reference samples and individuals with a Z-score < -5 for one of five nearest neighbors based on multidimensional scaling were removed; (c) relatedness check: duplicates were removed and a pair was removed if proportion identity by descent (PI_HAT) > 0.25. All quality control steps were performed using PLINK v1.9.

Candidate gene panels

UROseek panel [2]: *CDKN2A, ERBB2, FGFR3, HRAS, KRAS, MET, MLL, PIK3CA, TERT, TP53, VHL*

Panel by Le Goux *et al.* [3]: *BCL2L1, CCND1, CCNE1, E2F3, EGFR, ERBB2, ERBB3, ERCC2, FBXW7, FGFR3, FRS2, GDI2, HRAS, Ki67, MDM2, MDM4, MYC, NFE2L2, PABPC1, PAIP1, PIK3CA, PPARG, PRKCI, PVRL4, RXRA,* *SOX4, TACC3, YWHAZ, ZNF703*

Panel by Ward *et al*. [4]: *AKT1, BRAF, C3orf70, CDKN1A, CDKN2A, CREBBP, CTNNB1, ELF3, ERBB2, ERBB3, ERCC2, FBXW7, FGFR3, HRAS, KDM6A, KRAS, PIK3CA, RHOB, RXRA, SF3B1, TERT, TP53*, *NRA*

Definitions

Patients are included in the Nijmegen Bladder Cancer Study (NBCS) if (a) they are diagnosed with non-muscle invasive bladder cancer (NMIBC) and (b) they primarily underwent a transurethral resection of bladder tumour (TURBT), and (c) they were not diagnosed with concomitant upper tract urothelial carcinoma.

The date of progression was defined as the first date at which there was a transition from low-grade to high-grade disease, or an increase in T stage, N stage or M stage. Cystectomy for therapy-resistent or “uncontrollable” disease was also coded as progression.

Recurrence is defined as a new histologically confirmed bladder tumour following at least (a) one tumour-negative urethrocystoscopy (UCS) between the previous TURBT and the recurrence; or (b) a TURBT followed by a radical re-TURBT and thereafter a recurrence without negative UCS inbetween.

Histologically confirmed bladder tumours found in the bladder after cystectomy were coded as recurrence if the cystectomy was performed at least 5 months after the previous TURBT, otherwise it was seen as residual disease. Coagulations were also coded as recurrence, provided the coagulation took place at least 6 months after the previous TURBT. The coagulated lesions are assumed to be low grade stage Ta tumours.

The date of recurrence is determined as follows:

- In case the tumour seen by UCS is operatively removed by TURBT or biopsy, the date of removal marks the date of recurrence;
- In case the tumour seen by UCS is removed by fulguration or coagulation, the date of ablation marks the date of recurrence. In this case the tumours are assumed to be low grade Ta tumours;
- In case the tumour seen by UCS is not removed, the date of UCS marks the date of recurrence, unless this tumour is removed in a later instance.

First recurrences that are diagnosed within 4 months of primary TURBT are seen as residual disease and not coded as recurrence. We did not impose restrictions on the timing for subsequent recurrences.

Both for the analysis of progression and recurrences, the follow-up ended after the event of (a) cystectomy; (b) progression to MIBC; (c) last contact between patient and urologist; (d) End of follow-up NBCS (December 31^th^, 2011).

Model explanation and selection

*Cox proportional hazards model (Cox PH model)*

In the Cox model, the risk of experiencing a recurrence or progression at a given point in time is expressed by the hazard function. The hazard function incorporates a shared baseline hazard (denoted by λ_0_) for all patients, which is multiplied by an individual-specific risk factor exp(βX_i_). The hazard of individual i at time t is expressed as

λ_i_(t) = λ_0_(t)exp(βX_i_).

One can test for association between covariate X and recurrence-free survival by conducting inference on the ‘hazard ratio’ exp(β). The hazard ratio can be interpreted as the increased recurrence or progression risk for a unit increase of covariate X.

*Recurrent event model selection*

While the Cox PH model was initially designed for time-to-event data, it was later extended to be able to analyse recurrent event data. By including multiple recurrences per individual in analysis, several statistical challenges arise to appropriately model recurrence risks and handle correlated recurrence data within patients. It not possible to do recurrent event analysis on progression of NMIBC, since the event of progression can only occur once by definition. We will first discuss the model choices that are related to recurrent event data and methods to account for within-subject correlation. Afterwards, we will consider which recurrent event models are most adequate to analyse NMIBC recurrence data.

*Model options*

Two important facets of recurrent event models which make use of Cox regression are *risk intervals* and *risk sets*. Kelly and Lim [5] describe three commonly applied risk sets and risk intervals, which constitute the different recurrent event models shown in Table 1.

*Risk intervals* define the time scale on which recurrences occur. Three commonly applied risk interval types are:

- Gap time: time to recurrence is modelled as time since previous recurrence, essentially ‘resetting’ the clock after every new recurrence.
- Elapsed time: time to recurrence is modelled as time since a chosen time point, *e.g.* a primary diagnosis.
- Calendar time: time to recurrence is also modelled as time since a chosen time point. However, the recurrences are ordered. E.g.: a patient can only be at risk of the third recurrence when exactly two recurrences have previously taken place.

Figure 1 displays the different types of risk intervals in an individual with three recurrences. The time scale of calendar time and elapsed time models is relative to the primary event, whereas the time scale of gap time is relative to the previous recurrence.

A *risk set* defines which individuals are at risk of experiencing a recurrence on a given point in time. This risk set can be specific to a recurrence number (i.e. a restricted risk set), which results in a recurrence-specific baseline hazard. The risk set can also be common to all recurrences (i.e. an unrestricted risk set), resulting in a common baseline hazard for every recurrence. The third option is a semi-restricted risk set, in which case subjects who have experienced less than a number of *k*-1 recurrences may be at risk of the *k*th recurrence.

Three approaches have been proposed to account for within-subject correlation in inference: marginal, conditional and random effects. The marginal approach assumes that recurrences are independent within a subject, and uses a corrected ‘robust’ variance estimator for the hazard ratio. The conditional approach assumes that recurrences are independent within subjects, conditional on previous information on recurrence risk. In this case, the recurrence risk can be modelled by time-varying covariates such as the recurrence number. The random effects approach includes a frailty parameter per individual to model unobserved heterogeneity in individuals.

*Model choices to analyse the total NMIBC recurrence burden*

We will not use elapsed time models in analysis, because they are not realistic models for the bladder cancer data since the recurrences are not ordered. Semi-restricted risk sets are known to overestimate hazard ratios due to the ‘carry-over effect', therefore we will not consider this type of risk set (9). Since there is significant heterogeneity in recurrence risk of NMIBC patients which is only modelled by frailty models, the random effects approach is used in our recurrent event analysis.

By eliminating elapsed time models and models based on semi-restricted risk sets, four candidate models remain in Table 1. In order to determine whether a restricted risk set or an unrestricted risk set matches our data, baseline hazards of tumour recurrence were computed in both gap time and calendar time using a frailty model. These indicated that a common baseline hazard is appropriate on both time scales, since the common baseline hazard is similar to the event-specific baseline hazards. This leaves the Gap Time Unrestricted (GT-UR) model and the Andersen & Gill model as candidate models for analysis.

There are two types of recurrent event analyses: analysis based on *gap time* and analysis based on *calendar time*. Gap time models analyse risk of recurrence since the previous recurrence, whereas calendar time models analyse risk of recurrence since primary diagnosis. Hazard ratios in gap time models are interpreted as the increase of decrease of recurrence risk since the previous bladder tumour, whereas hazard ratio estimates in calendar time models are interpreted as effects on recurrence risk from primary transurethral resection of bladder tumour (TURBT) onwards. If one wishes to determine the recurrence risk on the total recurrence burden after the primary TURBT, a hazard ratio in calendar time will be more useful. If, on the other hand, one is interested in recurrence risk for the next recurrence after a recurrence, a hazard ratio in a gap time model will be more informative. Since we found the risk of the next recurrence to be clinically most relevant, we selected a gap time model, the GT-UR model, for the statistical analysis of tumour recurrence.

In the GT-UR model, the hazard function of individual *i* for recurrence *k* at time *t* is given by

λ_i,k_(t) = λ_0_(t - t_i,k-1_)u_i_exp(βX_i_),

Where u_i_ denotes the individual frailty, *k* denotes the recurrence number and t_i,k-1_ denotes the time of the k-1th recurrence of individual i since primary TURT, with the convention t_i,0_ = 0.

Model assumptions

The GT-UR model is based on the same assumptions as the CoxPH model. In addition, the GT-UR model assumes a constant effect of genetic variants on recurrence rate for all recurrences, although it is unknown whether genetic variants act similarly on the recurrence rate of all recurrence. Under this assumption, the recurrent event model gives more precise estimates on the recurrence risk compared to a CoxPH model. We assume that censoring is non informative. Ties of recurrence times are handled using Efron's correction for partial likelihood estimates. A lognormal frailty distribution was assumed.

Risk stratification in Figure 1

Prognostic risk groups were assessed using a modified version of EAU prognostic risk categories, as not all clinical data were available [6]. The difference with EAU prognostic risk categories is that risk factor ‘tumour diameter > 3 cm’ is replaced with ‘recurrent tumour’, as no information on tumour tumour diameter was available. In addition, we relaxed the condition that a low risk tumour should be a primary tumour. The definition of risk groups are included in Table 2.

| **Risk** **interval** | **Risk** **set** |  |  |
| --- | --- | --- | --- |
|  | **Unrestricted** | **Semi-restricted** | **Resctricted** |
| Gap time | GT-UR | Not possible | PWP-GT |
| Elapsed time | LWA | WLW | TT-R |
| Calendar time | Andersen and Gill | Possible | PWP-CT |

Table 1. Overview of recurrent event models, based on type of risk interval and risk set [5].

Table 2. Definitions of risk groups for Figure 1 in main article, obtained from EAU prognostic risk categories [6].

| **Risk Group** | |
| --- | --- |
| **Low risk** | - T1 LG^1^ tumours without CIS with no risk factors - Ta LG tumours without CIS with at most one risk factor |
| **Intermediate risk** | Tumours without CIS that are not included in low, high or very high-risk groups |
| **High risk** | - All T1 HG tumours no CIS, EXCEPT those included in the very high-risk group - All CIS tumours, EXCEPT those included in the very high-risk group |
|  | **Stage, grade with additional risk factors:**   - Ta LG or T1 G1, no CIS with all 3 factors - Ta HG or T1 HG, no CIS with at least 2 risk factors - T1 G2 no CIS with at least 1 risk factor |
| **Very high risk** | **Stage, grade with additional risk factors:**   - Ta HG and CIS with all 3 risk factors - T1 G2 and CIS with at least 2 factors - T1 HG and CIS with at least 1 factor - T1 HG no CIS with all 3 factors |

^1^LG denotes grade G1, G2 or G2a, HG denotes grade G2b or G3. Risk factors are: 1) Age > 70 years; 2) Multiple papillary tumours; and 3) Recurrent tumour (*i.e.* more than one per year on average).

**References**

1. Galesloot TE, Grotenhuis AJ, Kolev D, Aben KK, Bryan RT, Catto JWF, Cheng KK, Conroy S, Dyrskjot L, Fleshner NE, James ND, Lamy P, Lindskrog SV, Malats N, Mengual L, Verhaegh G, Zeegers MP, Kiemeney L, Vermeulen SH. Genome-wide Meta-analysis Identifies Novel Genes Associated with Recurrence and Progression in Non-muscle-invasive Bladder Cancer. Eur Urol Oncol. 2021.

2. Eich ML, Rodriguez Pena MDC, Springer SU, Taheri D, Tregnago AC, Salles DC, Bezerra SM, Cunha IW, Fujita K, Ertoy D, Bivalacqua TJ, Tomasetti C, Papadopoulos N, Kinzler KW, Vogelstein B, Netto GJ. Incidence and distribution of UroSEEK gene panel in a multi-institutional cohort of bladder urothelial carcinoma. Mod Pathol. 2019;32(10):1544-50.

3. Le Goux C, Vacher S, Schnitzler A, Barry Delongchamps N, Zerbib M, Peyromaure M, Sibony M, Allory Y, Bieche I, Damotte D, Pignot G. Assessment of prognostic implication of a panel of oncogenes in bladder cancer and identification of a 3-gene signature associated with recurrence and progression risk in non-muscle-invasive bladder cancer. Sci Rep. 2020;10(1):16641.

4. Ward DG, Gordon NS, Boucher RH, Pirrie SJ, Baxter L, Ott S, Silcock L, Whalley CM, Stockton JD, Beggs AD, Griffiths M, Abbotts B, Ijakipour H, Latheef FN, Robinson RA, White AJ, James ND, Zeegers MP, Cheng KK, Bryan RT. Targeted deep sequencing of urothelial bladder cancers and associated urinary DNA: a 23-gene panel with utility for non-invasive diagnosis and risk stratification. BJU Int. 2019;124(3):532-44.

5. Kelly PJ, Lim LL. Survival analysis for recurrent event data: an application to childhood infectious diseases. Stat Med. 2000;19(1):13-33.

6. Sylvester RJ, Rodriguez O, Hernandez V, Turturica D, Bauerova L, Bruins HM, Brundl J, van der Kwast TH, Brisuda A, Rubio-Briones J, Seles M, Hentschel AE, Kusuma VRM, Huebner N, Cotte J, Mertens LS, Volanis D, Cussenot O, Subiela Henriquez JD, de la Pena E, Pisano F, Pesl M, van der Heijden AG, Herdegen S, Zlotta AR, Hacek J, Calatrava A, Mannweiler S, Bosschieter J, Ashabere D, Haitel A, Cote JF, El Sheikh S, Lunelli L, Algaba F, Alemany I, Soria F, Runneboom W, Breyer J, Nieuwenhuijzen JA, Llorente C, Molinaro L, Hulsbergen-van de Kaa CA, Evert M, Kiemeney L, N'Dow J, Plass K, Capoun O, Soukup V, Dominguez-Escrig JL, Cohen D, Palou J, Gontero P, Burger M, Zigeuner R, Mostafid AH, Shariat SF, Roupret M, Comperat EM, Babjuk M, van Rhijn BWG. European Association of Urology (EAU) Prognostic Factor Risk Groups for Non-muscle-invasive Bladder Cancer (NMIBC) Incorporating the WHO 2004/2016 and WHO 1973 Classification Systems for Grade: An Update from the EAU NMIBC Guidelines Panel. Eur Urol. 2021;79(4):480-8.
